# The Relationship Between Medical Diagnoses, Risk Perceptions, and Social Distancing Compliance: An Analysis of Data from the Toledo Adolescent Relationships Study

**DOI:** 10.18061/ojph.v4i2.8352

**Published:** 2022-01-28

**Authors:** Ian Y. King, Wendy D. Manning, Monica A. Longmore, Peggy C. Giordano

**Affiliations:** 1 Department of Sociology, and Center for Family and Demographic Research, Bowling Green State University, Bowling Green, OH

**Keywords:** Physical health, Mental health, Compliance, Risk, COVID-19

## Abstract

**Background:**

The health belief model suggests that individuals’ beliefs affect behaviors associated with health. This study examined whether Ohioans’ pre-existing medical health diagnoses affected their belief about personal health risk and their compliance with social distancing during the coronavirus disease 2019 (COVID-19) pandemic. Prior research examining physical and mental diagnoses and social distancing compliance is nearly nonexistent. We examined whether physical and mental health diagnoses influenced individuals’ beliefs that their health is at risk and their adherence with social distancing guidelines.

**Methods:**

The study used longitudinal cohort data from the Toledo Adolescent Relationships Study (TARS) (n = 790), which surveyed Ohioans prior to and during the COVID-19 pandemic. Dependent variables included belief that an individual’s own health was at risk and social distancing compliance. Independent variables included physical and mental health diagnoses, pandemic-related factors (fear of COVID-19, political beliefs about the pandemic, friends social distance, family social distance, COVID-19 exposure), and sociodemographic variables (age, gender, race/ethnicity, educational level).

**Results:**

Individuals who had a pre-existing physical health diagnosis were more likely to believe that their personal health was at risk during the pandemic but were not more likely to comply with social distancing guidelines. In contrast, individuals who had a pre-existing mental health diagnosis were more compliant with social distancing guidelines but were not more likely to believe their personal health was at risk. Individuals who expressed greater fear of COVID-19 believed their health is more at risk than those who expressed lower levels of fear.

**Conclusion:**

Health considerations are important to account for in assessments of responses to the pandemic, beliefs about personal health risk, and social distancing behavior. Additional research is needed to understand the divergence in the findings regarding physical health, beliefs about personal health risk, and social distancing compliance. Further, research is needed to understand how mental health issues impact decision-making related to social distancing compliance.

## INTRODUCTION

In 2019, the coronavirus disease 2019 (COVID-19) was identified in the Wuhan province in China.^1^ As of October 2021, there have been 44 857 861 COVID-19 cases and 723 205 deaths in the United States due to this contagious disease.^2^ In Ohio alone, there have been 1 499 485 cases of COVID-19 as of October 2021 and 23 327 deaths.^3^ During the COVID-19 pandemic, various guidelines and mandates were put in place to ensure public health safety. In Ohio, examples included maintaining a 6-foot distance from others, known as social distancing, and wearing a mask when out in public. Even with the availability of COVID-19 vaccines, these public health measures continued to be recommended in Ohio. Moreover, it seems that following these guidelines would be especially important for those individuals most at risk for severe symptoms, such as individuals with prior health diagnoses, if they contract the virus.

According to the Centers for Disease Control and Prevention (CDC), risk for severe illness due to COVID-19 increases with the following pre-existing health diagnoses: cancer, chronic kidney disease, chronic lung disease, dementia, diabetes, down syndrome, heart conditions, AIDS/HIV, immunocompromised state, liver disease, obesity, sickle cell disease, solid organ or blood stem cell transplant, stroke, and substance use disorders.^4^ Because these health conditions put Ohioans most at risk for complications if they contract COVID-19, it is important to identify factors that affect compliance with public health recommendations and mandates among individuals with and without health diagnoses.

Further, although younger adults, compared with older adults, tend to have fewer physical health conditions, a significant proportion of young adults live with 1 or more physical health diagnoses. For example, among young adults in their 20s to early 40s, 2.9% have diabetes and 10.6% have high blood pressure,^5^ 8.5% have high cholesterol, and 4.7% have heart disease.^6^ According to the CDC, 2.3% of adults in their mid-20s to early 30s, suffer from hepatitis B, and .029% have AIDS/HIV.^7^ Among young adults, 8.0% live with asthma,^8^ and .9% have had a cancer diagnosis.^9^ Mental health concerns are also prevalent in this age group. Prior to the pandemic, 11.6% of individuals aged 25 to 35 years reported being diagnosed with anxiety, 15.2% reported diagnosed depression^10^, and 4.76% reported diagnosed ADD/ADHD.^11^ To date, however, there is little, if any, research regarding whether these pre-existing medical diagnoses contribute to individuals’ beliefs that their health is at risk due to COVID-19, and whether pre-existing medical diagnoses affect social distancing compliance. Studying this age group is of critical importance because in the United States individuals aged 20 to 49 years have accounted for much of the spread of the COVID-19 virus.^12^

There are varied reasons that individuals have provided for not following social distancing guidelines. In Italy, for example, individuals were less likely to comply if the duration of the stay-at-home order was longer than expected.^13^ Additionally, individuals who perceived the disease to be more deadly as evidenced by the number of people they know who have had COVID-19 reported a lower likelihood of social distancing compliance in what has been dubbed the “fatalism effect.”^14^ Thus, it appears that beliefs and expectations affect compliance.

Research on sociodemographic correlates of beliefs and behaviors during the pandemic has included gender, race/ethnicity, age, and economic background. More women than men have reported taking precautions to protect themselves from COVID-19.^15^ Possessing knowledge of COVID-19 has led to increased levels of social distancing for women, but not men.^16^ Women, compared with men, reported a greater sense of danger due to the pandemic.^17^ Socioeconomic background also has played a role in compliance in that those with higher socioeconomic status reported more instances of taking precautions against COVID-19.^15^ Yet, lower income prior to the pandemic was associated with a greater sense of danger due to the pandemic.^17^ Individuals with a high school education or lower have reported higher numbers of close contact (ie, less compliance with social distancing).^18^ Race/ethnicity was associated significantly with social distancing behaviors with Black respondents reporting higher compliance with social distancing.^6^ Some research has found that younger individuals, compared to older individuals, reported that they were less likely to go out in general; yet, individuals who were older than 70 years were less likely to have gone out the previous day compared with younger individuals.^18^ Older age also was associated with greater feelings of pandemic-related danger.^17^

Previous research has found that in the United States political party affiliation and political ideology have played a role in the degree to which individuals have complied with social distancing regulations. For example, individuals who resided in Republican counties were less compliant with stay-at-home orders than individuals who resided in Democratic counties.^19^ Additionally, individuals affiliated with the Democratic Party reported lower likelihoods of compliance when the stay-at-home order was issued by a Republican governor.^19^ Viewers of conservative media outlets, such as Fox News, tended to be less compliant with stay-at-home orders.^20^ Regarding beliefs about personal health risk, individuals who identify as Democrats reported higher levels of pessimism regarding health relative to individuals who identify as Republicans.^15^ Moreover, those who endorsed Donald Trump for US President were less likely to believe that they were at risk for COVID-19.^21^ In sum, in the United States, politics have influenced whether individuals believe they are at risk for COVID-19 and whether they followed social distancing guidelines.

Despite the serious health implications of COVID-19, there is a paucity of research on health diagnoses and compliance with social distancing guidelines^6^ or whether individuals believe that their health is at risk if they contract COVID-19. An important conceptual model for understanding health behaviors is the health belief model. This model posits that individuals will engage in health behaviors if they believe they are (1) more at risk for contracting a disease, (2) likely to experience more serious consequences for that disease, (3) able to access potential protection that could reduce susceptibility and/or severity of the disease, (4) able to benefit from potential protection efforts, and (5) certain that the benefits outweigh any barriers that could prevent the disease.^22^ As mentioned previously, several medical conditions can lead to serious health consequences from COVID-19.^4^ Following the health belief model, individuals with diagnosed medical conditions prior to the pandemic may be more likely to believe that they are at risk for serious consequences of COVID-19 and may be more compliant with social distancing guidelines. However, this may not be the case. As mentioned previously, politics can drive social distancing compliance^15,19,21^ and socioeconomic status can as well.15 Thus, it is imperative to examine beliefs about personal health risk and social distancing compliance separately because believing that one is at high risk may not necessarily influence greater compliance with social distancing recommendations and regulations.

The purpose of this study is to examine how physical and mental health diagnoses influence beliefs about personal health risk and social distancing compliance in Ohio utilizing a longitudinal cohort data set. Examining physical and mental health diagnoses from previous waves of data can work to prevent recall bias associated with cross-sectional studies.^6^ Longitudinal data allow more assurance of time order of the variables so we can be sure that the physical and mental health diagnoses occurred before the COVID-19 pandemic. We argued that beliefs about personal health risk may not influence social distancing compliance as the health belief model would suggest, as the COVID-19 pandemic has been increasingly politicized.^15,19,21^ The research questions assessed whether physical or mental health diagnoses affect: (1) belief about personal health risk, and (2) social distancing compliance. We expect that political beliefs^15,19,21^ and socioeconomic status15 would guide social distancing behaviors. Our first hypothesis states the following: Individuals with a physical or mental health diagnosis will be more likely to believe their personal health is at risk relative to individuals without a medical diagnosis. Our second hypothesis states the following: Individuals with a physical or mental health diagnosis will be more likely to follow the social distancing guidelines relative to individuals without a medical diagnosis.

Although the fatalism effect posits that respondents who know more people with COVID-19 will comply less with social distancing recommendations, this may not apply to individuals’ beliefs about their own risk.^14^ Based on prior work, COVID-19 fears, political beliefs about the severity of the pandemic, whether significant others follow social distancing guidelines, and likelihood of COVID-19 exposure will influence belief about personal health risk as well as adherence with social distancing.

The current study used longitudinal cohort data from the Toledo Adolescent Relationship Study (TARS) (n = 790), which surveyed Ohioans prior to and during the COVID-19 pandemic. Dependent variables included personal health risk belief and social distancing compliance. Independent variables included physical and mental health diagnoses, pandemic-related indicators (ie, fear of COVID-19, political beliefs about the pandemic, friends social distance, family social distance, COVID-19 exposure), and sociodemographic variables (age, gender, race/ethnicity, educational level).

## METHODS

### Setting and Design

This study used data from the Toledo Adolescent Relationship Study (TARS). The initial TARS sample was interviewed in 2000 and 2001 and consisted of a stratified random sample of 7th, 9th, and 11th graders from Lucas County, Ohio. According to US Census Bureau data, Lucas County is similar to national demographics regarding education, income, and race.^23^ The TARS data contains 7 waves of data with Wave 1 (2000–2001), Wave 6 (2019), and Wave 7 (2020) being utilized for this study. As such, the data were collected prior to and during the pandemic. Internal review board approval was received for each wave of data collection.

### Participants

The baseline sample included 1321 respondents aged 12 to 18 years. The most recent interview, Wave 7, included 822 respondents aged 31 to 38 years. The sampling frame was based on school rosters in Lucas County, Ohio, with an oversample of Black and Hispanic respondents. Rosters were available through Ohio’s Freedom of Information Act, and respondents did not have to attend school to participate in the study. Due to small sample sizes, we excluded respondents who reported their race as “other” (n = 18), or who were missing data on the dependent variables (n = 7). The final analytical sample is 790 respondents with 73.46% of the sample currently living in Ohio.

### Measures

#### Dependent Variables

Beliefs about personal health risk were collected at Wave 7 (during the pandemic). We asked how strongly respondents agreed or disagreed with the following: “I am at a high risk of becoming infected.” The scale ranged from (1) strongly disagree to (5) strongly agree.

Social distancing compliance was collected at Wave 7 and is a self-developed 6-item summed scale. Respondents were asked how often they did the following when the social distancing guidelines were suggested: (1) “increase physical space between you and others (six feet is recommended) to avoid spreading illness,” (2) “stay home to avoid spreading illness,” (3) “go to grocery store or pharmacy,” (reversed) (4) “go to a workplace that requires contact with others,” (reversed) (5) “hang out or spend time with friends (not living with you),” (reversed) and (6) “hang out or spend time with family (not living with you)” (reversed). The scale ranged from (1) never to (5) as much as possible (α = .70).

#### Independent Variables

Physical health diagnoses were collected at Wave 6, prior to the pandemic, and were measured by asking whether respondents were told by a doctor, nurse, or other health care provider that they have “cancer, lymphoma, or leukemia,” “high cholesterol, triglycerides, or lipids,” “high blood pressure or hypertension,” “high blood sugar or diabetes,” “heart disease or heart failure,” “asthma,” “chronic bronchitis or emphysema,” “epilepsy or another seizure disorder,” “hepatitis B or C,” “sleep apnea,” “chronic kidney disease or kidney failure,” “blood clot, stroke, or mini stroke,” “HIV/AIDS,” or “a sexually transmitted disease such as genital herpes, warts, chlamydia, HPV, gonorrhea, or syphilis.”^24^ Responses were (0) no and (1) yes.

Mental health diagnoses were collected at Wave 6 and were measured by asking whether respondents were told by a doctor, nurse, or other health care provider that they have “depression,” “post-traumatic stress disorder or PTSD,” “anxiety or panic disorder,” or “attention problems or ADD or ADHD.”^24^ Responses were (0) no and (1) yes.

#### COVID-19 Indicators

Fear of COVID-19 was a self-developed 3-item summed scale collected at Wave 7. We asked how often during the pandemic did respondents experience the following: (1) “Worried you might have contracted the virus,” (2) “Worried one or more of family might contact COVID-19,” and (3) “Listened to news or read social media about COVID-19 developments.” Responses included (1) never to (5) very often (α = .71).

Political beliefs were a self-developed 2-item summed scale collected at Wave 7, which asked how strongly respondents agreed or disagreed with the following: (1) “Politicians, the news, and social media have exaggerated the risk,” and (2) “Government should not tell me what to do.” Responses included (1) strongly disagree to (5) strongly agree (α = .71).

Friends social distance compliance was measured at Wave 7 with the following: “How many of your friends and acquaintances practice social distancing?” Responses included (1) none to (5) all.

Family social distance compliance was measured at Wave 7 with the following: “How many of your family members practice social distancing?” Responses included (1) none to (5) all.

Exposure to COVID-19 was a self-developed 2-item summated scale. We asked the following: (1) “Do you personally know someone who has/had the virus,” and (2) “Do you know someone who is in a job that puts them at higher risk for exposure to COVID-19?” The scale responses were (0) no and (1) yes (α = .46).

#### Sociodemographic Indicators

Age is measured at the Wave 7 interview. Respondents were, on average, age 34, with a range of 31 to 38 years of age. Gender is measured at Wave 1, with male as the comparison. Race/ethnicity is measured at Wave 1, and included non-Hispanic White (reference), non-Hispanic Black, and Hispanic. Educational attainment, measured at Wave 6, included high school or less (reference), some college, and college graduate. Month of interview indicated when respondents completed the interview ranging from 6 (June) to 10 (October/November).

### Statistical Analyses

We examined descriptive statistics for all variables (Table 1). Next, we estimated belief about personal health risk with Ordinary Least Squares (OLS) regression models (Table 2). Model 1 regressed personal health risk onto physical health diagnosis and mental health diagnosis. Model 2 regressed belief about personal health risk onto physical health diagnosis, mental health diagnosis, and the COVID-19 variables. Model 3 regressed health belief onto physical health diagnosis, mental health diagnosis, and the socio-demographic variables. Model 4 regressed belief about personal health risk onto physical health diagnosis, mental health diagnosis, the COVID-19 variables, and the sociodemographic variables. Finally, we examined social distancing compliance in terms of physical and mental health diagnoses with a series of OLS regression models (Table 3). Model 1 regressed compliance on physical health diagnosis and mental health diagnosis. Model 2 regressed compliance on physical health diagnosis, mental health diagnosis, and the COVID-19 variables. Model 3 regressed compliance on physical health diagnosis, mental health diagnosis, and the socio-demographic variables. Model 4 regressed compliance on physical health diagnosis, mental health diagnosis, the COVID-19 variables, and the sociodemographic variables. Interview month is included but not presented in the tables.

## RESULTS

The mean value of belief about personal health risk is 2.69, which represents a midpoint on a scale ranging from 1 to 5 (Table 1). The average social distancing score was 19.65, indicating that most respondents responded to values above the midpoint. Regarding the key independent variables, over half (50.82%) reported a physical health diagnosis, and 43.65% reported a mental health diagnosis. The mean score for fear of COVID-19 was 9.62 indicating that most respondents responded to values above the midpoint. The mean score on conservative political beliefs was 5.93 indicating values just below the midpoint of the scale, which ranged between 2 and 10. Friends who social distance and family who social distance averaged above the midpoint, 3.64 and 3.72, respectively, indicating values just above the midpoint of the scale between 1 and 5. Exposure to COVID-19 was low with a mean score of 1.43 indicating values just above the midpoint of the scale between 0 and 2.

Respondents’ mean age was 34.10. Nearly 60% of the sample was female. One-fifth (20.00%) of the sample was Black, 11.07% Hispanic and two-thirds (68.93%) White. About 18.49% of respondents have a high school degree or less, 42.14% reported some college, and 39.37% reported a college degree.

In Model 1 (Table 2), physical health was associated positively with belief about personal health risk. Physical health diagnosis is positively related to both personal health belief risk and the health belief risks of others. Mental health was not significantly related to belief about personal health risk in Model 1, and this was true even when physical health was not included in the model (not shown). In Model 2, the relationship between physical health and belief about health risk remained significant with the inclusion of the COVID-19 variables. Individuals who expressed greater fear of COVID-19 believed their personal health is more at risk than those who expressed lower levels of fear. Model 3 examined how physical health diagnosis, mental health diagnosis, and the sociodemographic variables influenced belief about personal health risk. The significant relationship between physical health diagnosis remained the same as in Models 1 and 2. Women, compared with men, were more likely to believe their health was at risk. Black, compared with White, respondents were more likely to believe their health was at risk. Individuals with a college degree, compared to those without a degree, were less likely to believe their health was at risk. Model 4 included the full set of covariates and showed how physical health diagnosis, mental health diagnosis, the COVID-19 variables, and the sociodemographic variables influenced health beliefs. The significant relationship between physical health diagnosis and belief about personal health risk remained the same in all models. Fear of COVID-19, family social distance, exposure to COVID-19, gender, and possessing a college degree remained significant. Those with a college degree or more were significantly less likely to believe that their health is at risk.

In Model 1 (Table 3), mental health diagnosis was found to be positively associated with social distancing compliance. Those with a mental health diagnosis were more likely to adhere to social distance guidelines compared to those without a mental health diagnosis. Physical health diagnosis was not associated with social distancing compliance and was not associated with compliance in a model without the mental health indicator (not shown). In Model 2, the relationship between mental health and social distancing compliance remained significant with the inclusion of the COVID-19 variables. Those who expressed greater fear of COVID-19 were more likely to social distance than those who expressed lower levels of fear. Individuals who expressed conservative political beliefs were less likely to social distance than individuals who expressed liberal political beliefs. Individuals who have friends and family who social distance were more likely to social distance than individuals whose friends and family did not. Model 3 examined how physical health diagnosis, mental health diagnosis, and the sociodemographic variables influenced social distancing compliance. The significant relationship between mental health diagnosis and social distancing compliance was similar as reported in Models 1 and 2. Further, older individuals, compared to younger individuals, were less likely to social distance. Women, compared with men, were more likely to social distance. Individuals with a college degree or more were more likely to social distance than those without a college degree. Model 4 examined how physical health diagnosis, mental health diagnosis, the COVID-19 variables, and the sociodemographic variables affected social distancing compliance. The significant relationship between mental health diagnosis and social distancing compliance remained across all models. Fear of COVID-19, conservative political beliefs, friends and family social distancing, age, and gender all remained statistically significant. Educational attainment was not associated with social distancing once the full set of covariates were included in the model.

## DISCUSSION

Using longitudinal cohort data collected in Ohio the current study examined whether physical health and mental health diagnoses were associated with beliefs about personal health risk and social distancing compliance. We found that individuals who were diagnosed with a physical health problem prior to the pandemic were more likely to believe that their health was at risk during the pandemic. Despite this finding, Ohioans with a physical health diagnosis were not more likely to comply with social distancing guidelines. Individuals who had received a mental health diagnosis from a doctor or other professional prior to the pandemic were more likely to comply with social distancing recommendations than individuals who did not have a mental health diagnosis. Individuals with a prior mental health diagnosis, however, were not more likely to believe their health was at risk.

The current study adds to a limited body of research on physical and mental health issues and social distancing behaviors. Consistent with a recent study by Papageorge and colleagues,^6^ results do not indicate a significant relationship between physical health and social distancing, yet the findings indicate that individuals who have a physical health diagnosis believe they are more at risk for COVID-19 even as they do not report greater compliance with social distancing.

Thus, complicating the basic tenets of the health belief model, these individuals recognize that they are at risk but are not more likely to take the actions needed to protect themselves from the virus. This could reflect structural or social impediments to effective social distancing, or attitudes, such as fatalism^5^ or political beliefs^15,19,21^ that may play a role. If the fatalism effect is at play here, these results suggest that it is not just the risk of others that influences social distancing compliance, but their own risk may cause less social distancing compliance. Future research should examine how health belief risk affects social distancing compliance. Conversely, individuals who had a previous mental health diagnosis are more likely to social distance, even as they indicate that they are not at greater risk for COVID-19. This is consistent with recent CDC findings underscoring that none of the mental health indicators contribute to having a higher risk of contracting COVID-19.^4^ Some mental health conditions may be associated with a more general decrease in the desire to socialize, and conditions such as agoraphobia, in particular,^25,26^ relate to a fear of leaving home. Both anxiety and depression may be linked to an increase in other types of ‘fears’ resulting in a heightened sensitivity to the issue of COVID-19 and resulting desire to comply fully with the social distancing recommendations.

The results of this study point to the need to examine the divergence in findings; those with physical health diagnoses recognized their risks but were not more likely to comply. Although we find that the associations between health and pandemic-related beliefs and behaviors are not explained by COVID-19 indicators or socio-demographic measures, future research needs to consider the type of health condition or severity of the health condition.

This study, however, is not without limitations. First, the TARS sample is concentrated around Lucas County, Ohio. Due to the local nature of the data, it is not possible to generalize to the entire population of either Ohio or the United States. Nevertheless, the characteristics of Toledo, Ohio, and the surrounding area are similar to those of other Ohio regions in terms of racial diversity and age^27^ and to national demographics in terms of education, income, and racial diversity.^23^ In addition, this study does not account for degree of severity for individual diagnoses as the health diagnoses measures are dichotomous variables. It may be that individuals suffer from varying degrees of their diagnosis. It is also possible that although individuals may have physical health diagnoses, their relatively young ages may play a role. Finally, this study does not examine underlying motivations for social distancing or beliefs about personal health risk. Further research determining how the pandemic has shaped beliefs and behaviors is warranted. Despite these limitations, this study makes contributions to the literature on social distancing compliance and beliefs about health risk.

This study contributes to the literature on social distancing compliance and beliefs about personal health risk in 2 key ways. Although previous research has focused mostly on gender,^15^ the length of the pandemic,^13^ and politics,^6,15,19^ this study focused on the physical and mental health circumstances of a large, heterogeneous sample. Receiving a diagnosis from a doctor or health care provider may be a more accurate indicator of current health of the respondent than self-reported physical health or mental health. Additionally, TARS is a longitudinal study, whereas many recent studies on COVID-19 are cross-sectional so causality cannot be established. Other recent studies have relied on convenience samples, so generalizability is questionable, or are based on retrospective questions that are subject to recall bias.6 With longitudinal data, we were able to examine how earlier medical diagnoses impacted current social distancing compliance and beliefs about personal health risk.

## PUBLIC HEALTH IMPLICATIONS

Although health beliefs are important for understanding compliance with various public health recommendations, the current study describes a disjuncture between beliefs and action that warrants greater attention by researchers and practitioners. Those adults in their mid-30s who had received a physical health diagnosis well understood that they were at increased risk but did not take the efficacious actions that corresponded to those beliefs. Conversely, the respondents who had received mental health diagnoses did not believe they were at heightened risk (consistent with CDC findings indicating no increased risk4), but nevertheless were more likely to comply than those without such diagnoses. This suggests the need for researchers to continue to investigate mechanisms underlying not only the association between beliefs and action but differences between general viewpoints and the process of making changes in basic patterns of social behavior. Recognizing the way individuals are positioned economically, politically, and socially may affect the nature of beliefs, compliance itself, and these disjunctures. Public health messages should be sensitive to these complex influences, and to variability in life circumstances as reflected in physical and mental health problems.

There have been 1 089 357 cases of COVID-19 in Ohio as of May 2021 and there have been 19 528 deaths due to COVID-19 as of May 2021.^3^ The daily COVID-19 cases in Ohio have been between 1000 and 5000 from January until April of 2021, with numbers decreasing in May.^3^ Although signs of improvement are encouraging, understanding the dynamics involved in social distancing is important as this can be an effective strategy in the event of future outbreaks. It is well-documented that young adults are not the most vulnerable age group in terms of general risk, but those with health problems constitute an important exception to this general finding.

## Figures and Tables

**Table 1.** Means/Percentages and Standard Deviations of Dependent Variable, Independent Variables, and Control Variables (n=790)

	%/Mean (SD)	Min	Max
**Dependent variables**			
Belief about health risk	2.69	1	5
Social distancing compliance	19.65	5	25
**Health diagnosis**			
Physical health diagnosis	50.82%	0	1
Mental health diagnosis	43.65%	0	1
**COVID-19 variables**			
Fear of COVID-19	9.62	3	15
Political beliefs	5.93	2	10
Friends social distance	3.64	1	5
Family social distance	3.72	1	5
Exposure to COVID-19	1.43	0	2
**Sociodemographic variables**			
Age	34.10	31	38
Gender			
Male	40.35%	0	1
Female	59.65%	0	1
Race/Ethnicity			
White	68.93%	0	1
Black	20.00%	0	1
Hispanic	11.07%	0	1
Educational attainment			
High school or less	18.49%	0	1
Some college	42.14%	0	1
College degree or more	39.37%	0	1
Month of interview completion			
June	36.23%	0	1
July	24.91%	0	1
August	18.24%	0	1
September	12.70%	0	1
October/November	7.92%	0	1

Source: Toledo Adolescent Relationship Study (TARS) 2001–2020

Dependent variables collected in seventh interview (2020)

Independent Variables collected at first, sixth, and seventh interviews (2001–2020)

**Table 2.** OLS Regression Models Estimating Personal Health Risk (n=793)

	Model 1			Model 2			Model 3			Model 4		
	b	se		b	se		b	se		b	se	
Intercept	2.49	.09	***	1.06	.35	**	1.62	.87		−.11	.85	
**Health diagnosis**												
Physical health diagnosis	.24	.09	**	.24	.08	**	.23	.09	*	.22	.08	**
Mental health diagnosis	.09	.09		-.02	.09		.04	.09		−.05	.09	
**COVID-19 variables**												
Fear of COVID-19	---	---		.15	.02	***	---	---		.16	.02	***
Political beliefs	---	---		.01	.02		---	---		−.01	.02	
Friends social distance	---	---		.04	.07		---	---		.06	.06	
Family social distance	---	---		-.12	.06	*	---	---		−.14	.06	*
Exposure to COVID-19	---	---		.15	.07	*	---	---		.17	.07	**
**Sociodemographic variables**												
Age	---	---		---	---		.03	.03		.05	.02	
Gender												
(Male)												
Female	---	---		---	---		.11	.09	***	.05	.09	
Race/Ethnicity												
(White)												
Black	---	---		---	---		.01	.12	**	−.06	.11	
Hispanic	---	---		---	---		−.09	.14		−.21	.13	
Educational attainment												
(High school or less)												
Some college	---	---		---	---		−.10	.12		−.15	.12	
College degree or more	---	---		---	---		−.30	.18	*	−.52	.12	***

Source: Toledo Adolescent Relationship Study (TARS) 2001–2020

Notes:

* p ≤ .05

** p ≤.01

*** p ≤ .001. Reference category in parentheses. Month included but not shown.

**Table 3.** OLS Regression Models Estimating Social Distancing Compliance (n=790)

	Model 1			Model 2			Model 3			Model 4		
	b	se		b	se		b	se		b	se	
Intercept	19.88	.23	***	16.98	.82	***	23.12	2.14	***	21.28	2.00	***
**Health diagnosis**												
Physical health diagnosis	−15	.22		−.20	.20		−.09	.22		−.16	.20	
Mental health diagnosis	.67	.23	**	.64	.20	***	.50	.23	*	.52	.20	*
**COVID-19 variables**												
Fear of COVID-19	---	---		.19	.04	***	---	---		.18	.04	***
Political beliefs	---	---		−.42	.05	***	---	---		−.38	.05	***
Friends social distance	---	---		.61	.15	***	---	---		.57	.15	***
Family social distance	---	---		.36	.14	*	---	---		.39	.14	**
Exposure to COVID-19	---	---		.01	.16		---	---		.00	.16	
**Sociodemographic variables**												
Age	---	---		---	---		−.13	.06	*	−.14	.06	**
Gender												
(Male)												
Female	---	---		---	---		1.16	.22	***	.67	.20	***
Race/Ethnicity												
(White)												
Black	---	---		---	---		.40	.29		.02	.26	
Hispanic	---	---		---	---		.39	.35		.21	.32	
Educational attainment												
(High school or less)												
Some college	---	---		---	---		−.07	.30		−.12	.27	
College degree or more	---	---		---	---		.93	.31	**	.15	.29	

Source: Toledo Adolescent Relationship Study (TARS) 2001–2020

Notes:

* p ≤ .05

** p ≤.01

*** p ≤ .001. Reference category in parentheses. Month included but not shown.

## References

[1] About COVID-19. Centers for Disease Control and Prevention. September 1, 2020. Accessed April 15, 2021. https://www.cdc.gov/coronavirus/2019-ncov/cdcresponse/about-COVID-19.html

[2] CDC COVID Data Tracker. Centers for Disease Control and Prevention. 2021. Accessed October 19, 2021. https://covid.cdc.gov/covid-data-tracker/#datatracker-home

[3] COVID-19 Dashboard. Ohio Department of Health. 2021. Accessed October 19, 2021. https://coronavirus.ohio.gov/wps/portal/gov/covid-19/dashboards

[4] Medical Conditions. Centers for Disease Control and Prevention. October 14, 2021. Accessed November 1, 2021. https://www.cdc.gov/coronavirus/2019-ncov/need-extra-precautions/people-with-medical-conditions.html

[5] Harris KM. An integrative approach to health. Demography. 2010;47 (1):1–22. 10.1353/dem.0.0091 20355681 PMC3000007

[6] Papageorge NW, Zahn MV, Belot M, Socio-demographic factors associated with self-protecting behavior during the Covid-19 pandemic. J Popul Econ. 2021;34:691–738. 10.1007/s00148-020-00818-x 33462529 PMC7807230

[7] Hepatitis B Surveillance in the United States, 2018. Centers for Disease Control and Prevention. Published July 27, 2020. Accessed May 15, 2021. https://www.cdc.gov/hepatitis/statistics/2018surveillance/HepB.htm

[8] Arispe IE, Gindi RM, Madans JH. National Center for Health Statistics. Health, United States, 2019. Hyattsville, MD. 2021. 10.15620/cdc:100685 33818995

[9] Miller HV, Barnes JC, Beaver KM. Self-control and health outcomes in a nationally representative sample. Am J Health Behav. 2011;35(1):15–27. 10.5993/ajhb.35.1.2 20950155

[10] Tietjen GE, Karmakar M, Amialchuk AA. Emotional abuse history and migraine among young adults: A retrospective cross-sectional analysis of the add health dataset. Headache. 2016;57(1):45–59. 10.1111/head.12994 27917478

[11] Dunn EC, Busso DS, Raffeld MR, Does developmental timing of exposure to child maltreatment predict memory performance in adult-hood? Results from a large, population-based sample. Child Abuse Negl. 2016;51:181–191. 10.1016/j.chiabu.2015.10.014 26585216 PMC4713298

[12] Monod M, Blenkinsop A, Xi X, Report 32:Age groups that sustain resurging COVID-19 epidemics in the united states. Science. 2021;371:1336. 10.1101/2020.09.18.20197376 PMC8101272 33531384

[13] Briscese G, Lacetera N, Macis M, Tonin M. Expectations, reference points, and compliance with COVID-19 social distancing measures. NBER Work Pap Ser. 2020:1–38. 10.3386/w26916 PMC9870805 36714370

[14] Akesson J, Ashworth-Hayes S, Hahn R, Metcalfe R, Rasooly I. Fatalism, beliefs, and behaviors during the COVID-19 pandemic. NBER Work Pap Ser. 2020:1–56. 10.3386/w27245 PMC9161200 35669928

[15] Fan Y, Orhun AY, Turjeman D. Heterogeneous actions, beliefs, constraints and risk tolerance during the COVID-19 pandemic. NBER Work Pap Ser. 2020:1–41. 10.3386/w27211

[16] Iorfa SK, Ottu IFA, Oguntayo R, COVID-19 knowledge, risk perception and precautionary behaviour among Nigerians: A moderated mediation approach. Front Psychol. 2020;11:1–10. 10.37473/dac/10.1101/2020.05.20.20104786 33329202 PMC7714760

[17] Kimhi S, Marciano H, Eshel Y, Adini B. (2020). Resilience and demographic characteristics predicting distress during the COVID-19 crisis. Soc Sci Med. 2020;265:1–6. 10.1016/j.socscimed.2020.113389 PMC7518838 33039732

[18] Canning D, Karra M, Dayalu R, Guo M, Bloom DE. The association between age, COVID-19 symptoms, and social distancing behavior in the United States. medRxiv. 2020. 10.1101/2020.04.19.20065219

[19] Painter M, Qiu T. Political beliefs affect compliance with COVID-19 social distancing orders. SSRN Electronic Journal. 2020. 10.2139/ssrn.3569098

[20] Simonov A, Sacher S, Dubé J-P, Biswas S. The persuasive effect of Fox News: Non-compliance with social distancing during the COVID-19 pandemic. NBER Work Pap Ser. 2020:1–70. 10.3386/w27237

[21] Barrios JM, Hochber Y. Risk perception through the lens of politics in the time of the covid-19 pandemic. NBER Work Pap Ser. 2020:1–26. 10.3386/w27008

[22] Skinner CS, Tiro J, Champion VL, Rimer BK, Viswanath K. The health belief model. In: Glanz K, ed. Health Behavior: Theory, Research, and Practice. San Francisco, CA: Jossey-Bass; 2015:75–94.

[23] Warner TD, Giordano PC, Manning WD, Longmore MA. Everybody’s doin’ it (right?): Neighborhood norms and sexual activity in adolescence. Soc Sci Res. 2011:40(6):1676–1690. 10.1016/j.ssresearch.2011.06.009 22427712 PMC3302666

[24] Jensen TM, Harris KM. A longitudinal analysis of stepfamily relationship quality and adolescent physical health. J Adolesc Health. 2017;61 (4):486–492. 10.1016/j.jadohealth.2017.04.015 28732714 PMC5646364

[25] Turgeon L, Marchand A, Dupuis G. Clinical features in panic disorder with agoraphobia: A comparison of men and women. J Anxiety Disord. 1998;12(6):539–553. 10.1016/S0887-6185(98)00031-0 9879034

[26] Agoraphobia. Mayo Clinic. November 18, 2017. Accessed May 17, 2021. https://www.mayoclinic.org/diseases-conditions/agoraphobia/symptoms-causes/syc-20355987

[27] Ohio Population Climbs 2.3% From 2010 to 2020. United State Census Bureau. August 25, 2021. Accessed October 10, 2021. https://www.census.gov/library/stories/state-by-state/ohio-population-change-between-census-decade.html

